# A comprehensive database of quality-rated fossil ages for Sahul’s Quaternary vertebrates

**DOI:** 10.1038/sdata.2016.53

**Published:** 2016-07-19

**Authors:** Marta Rodríguez-Rey, Salvador Herrando-Pérez, Barry W. Brook, Frédérik Saltré, John Alroy, Nicholas Beeton, Michael I. Bird, Alan Cooper, Richard Gillespie, Zenobia Jacobs, Christopher N. Johnson, Gifford H. Miller, Gavin J. Prideaux, Richard G. Roberts, Chris S.M. Turney, Corey J.A. Bradshaw

**Affiliations:** 1 School of Biological Sciences, University of Adelaide, Adelaide, South Australia 5005, Australia; 2 Department of Biogeography and Global Change, National Museum of Natural Sciences—Spanish Research Council (CSIC), c/José Gutiérrez Abascal 2, 28006 Madrid, Spain; 3 School of Biological Sciences, University of Tasmania, Private Bag 55, Hobart, Tasmania 7001, Australia; 4 Department of Biological Sciences, Macquarie University, New South Wales 2109, Australia; 5 College of Science, Technology and Engineering and Centre for Tropical Environmental and Sustainability Studies, College of Science Technology and Engineering, James Cook University, Cairns, Queensland 4870, Australia; 6 Centre for Archaeological Science, School of Earth and Environmental Sciences, University of Wollongong, New South Wales 2522, Australia; 7 Archaeology & Natural History, School of Culture, History & Language, Australian National University, Canberra, Australian Capital Territory 0200, Australia; 8 Institute of Arctic and Alpine Research, Geological Sciences, University of Colorado, Boulder, Colorado 80309-0450 USA; 9 School of Biological Sciences, Flinders University, Bedford Park, South Australia 5042, Australia; 10 Climate Change Research Centre, School of Biological, Earth and Environmental Sciences, University of New South Wales, New South Wales 2052, Australia

**Keywords:** Palaeontology, Palaeoecology, Ecological modelling

## Abstract

The study of palaeo-chronologies using fossil data provides evidence for past ecological and evolutionary processes, and is therefore useful for predicting patterns and impacts of future environmental change. However, the robustness of inferences made from fossil ages relies heavily on both the quantity and quality of available data. We compiled Quaternary non-human vertebrate fossil ages from Sahul published up to 2013. This, the *FosSahul* database, includes 9,302 fossil records from 363 deposits, for a total of 478 species within 215 genera, of which 27 are from extinct and extant megafaunal species (2,559 records). We also provide a rating of reliability of individual absolute age based on the dating protocols and association between the dated materials and the fossil remains. Our proposed rating system identified 2,422 records with high-quality ages (i.e., a reduction of 74%). There are many applications of the database, including disentangling the confounding influences of hypothetical extinction drivers, better spatial distribution estimates of species relative to palaeo-climates, and potentially identifying new areas for fossil discovery.

## Background & Summary

Fossils and geo-historical data have received high research interest since the 1980s to track trends (e.g., diversification and extinction) in the history of life^1^. New disciplines such as palaeo-ecoinformatics^2^ and conservation palaeo-biology^3^ have emerged as a result of the compilation of such data, providing crucial insights into long-term ecological and genetic processes, including evidence of the impact of past environmental changes^4^. Testing such eco-evolutionary phenomena is strongly time-dependent, so the entire range of archaeological and palaeontological research disciplines benefits from the improvement of fossil-dating techniques and the availability of high-quality chronologies for species occurrences.

The ever-increasing number of scientifically described fossil records has resulted in a burgeoning number of databases that compile dated fossils of vertebrate species across various spatio-temporal scales. These include *inter alia* the pioneering FAUNMAP (www.ucmp.berkeley.edu/faunmap), MioMap (www.ucmp.berkeley.edu/miomap), the *Paleobiology Database* (paleobiodb.org), *Neotoma Paleoecology Database* (www.neotomadb.org), New Zealand’s *Fossil Record Electronic Database* (FRED: www.fred.org.nz), and the *New and Old Worlds* (NOW) *Database of Fossil Mammals* (www.helsinki.fi/science/now). In the Sahul region (the combined landmass of Australia and New Guinea, including the areas of continental shelf exposed at lower sea levels), *The Atlas of Prehistoric Australia* (APA: apa.ala.org.au) is the only database that includes fossil occurrences and their relative ages for the Quaternary period (the last 2.6 million years). Thus far, attempts to catalogue absolute ages of vertebrate fossils in Australasia have been restricted to *Homo sapiens* (*AustArch*: http://dx.doi.org/10.5284/1027216)^5,6^.

The nineteenth-century anatomist Sir Richard Owen^7^ was the first to describe the existence of extinct large marsupials in Sahul, followed soon thereafter by others identifying new Australian species from fossils^8,9^. It was not until around 1950, however, that the first absolute dating of these fossils became possible with the development of radiocarbon dating^10^. Since the advent of such dating techniques, palaeontologists and archaeologists have published a growing volume of dated fossil species occurrences, most of which are described in independent scientific papers scattered throughout the literature.

The compilation of fossil descriptions and age estimates in databases has traditionally focussed on maximizing the quantity of fossil ages, with little attention specifically to their reliability (quality). However, unreliable (i.e., uncertain or incorrect) ages can potentially lead to erroneous conclusions regarding the chronology of environmental processes; for instance, there is still substantial disagreement and long-standing debate on the relative role of different drivers of extinction of the Late Pleistocene megafauna in Sahul, and these disputes are fuelled by reliance by some authors on ages that some consider to be erroneous^11–13^. To improve our capacity to disentangle the potentially confounding roles of different extinction processes, we present *FosSahul* (Data Citation 1), the first database of absolute ages of nonhuman (mostly terrestrial) vertebrate fossils (including all megafauna species). *FosSahul* is unique because it includes ratings of reliability (based on reference^11^) allocated to each fossil age and comprehensive metadata (georeferenced locations, dated materials, stratigraphic contexts) from the Pleistocene to the present in the Sahul region (from 1 Ma to present), current as of October 2013. The database will be updated as newly dated specimens and material are published.

## Methods

**Figure 1:**
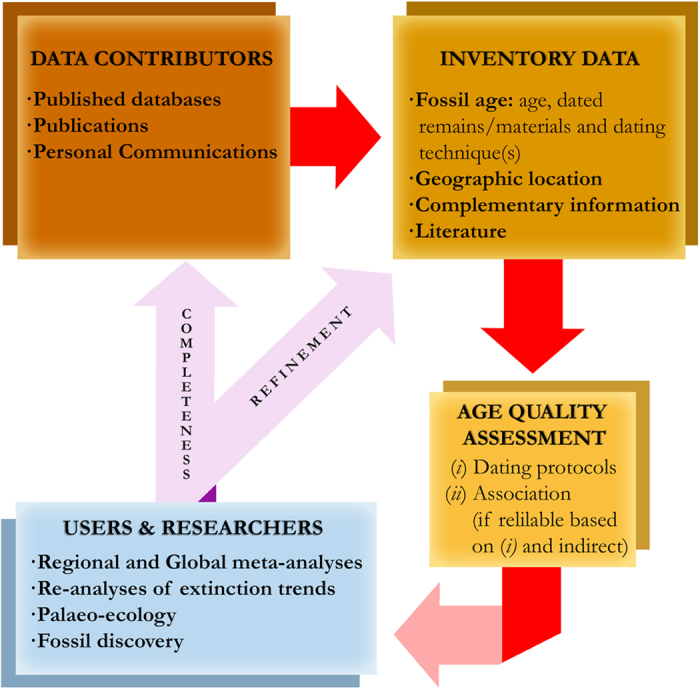
Flow diagram of the construction of the *FosSahul* database and future improvements.

Our database comprises Pleistocene to Holocene ages for fossils of terrestrial and freshwater vertebrates (non-human mammals, birds, reptiles and amphibians) from the Sahul region, published up to October 2013. The main elements of the database are described in Fig. 1, and below.

### Literature search

We accessed fossil ages in three steps: we (*i*) collated age data from the primary literature (‘core papers’) by searching within article titles, abstracts and keywords in ISI *Web of Science*® (webofscience.com) using the search terms—((‘Late-Pleistocene’ or ‘Holocene’) and (‘Sahul’ or ‘Australia’ or ‘New Guinea’) and ‘megafauna’); (*ii*) retrieved additional ages by cross-referencing and accessing literature cited in the core papers; and (*iii*) scrutinized the full set of literature sources (primary and secondary archaeological literature, including cross-references) in the *AustArch* database (http://dx.doi.org/10.5284/1027216) of *Homo* fossils^14^ for fauna records associated with dated archaeological information. Thus, we included non-megafauna vertebrate fossils only when published along with megafauna and archaeological remains. Throughout and where possible, we contacted the authors responsible for publishing many of the fossil ages (see Acknowledgements) when clarification was required (e.g., stratigraphic context, laboratory labels).

### Data compilation

For each species record, we collated the age estimate(s) and associated metadata classified into six fields (and several sub-fields) including Linnaean classification of species, ratings of age reliability, geographical location, contextual information and literature sources (Table 1 (available online only)).

#### Linnaean classification

We classified species into six taxonomic levels (*Order*, *Class*, *Infra-Class*, *Family*, *Genus*, *Species*) and two categories (‘Status’ and ‘Megafauna’) that differentiate whether they are extant or extinct and belonged to the megafauna assemblage (i.e., species with a body mass >44 kg or approximately >100 lbs). We checked for concordance between Linnaean classifications of individual species across publications in the *Paleobiology Database* (paleobiodb.org), the *Global Biodiversity Information Facility* (GBIF; www.gbif.org) and the International Union for the Conservation of Nature’s (IUCN) *Red List of Threatened Species* (www.iucnredlist.org), and the latest published taxonomic revisions. When only Genus, Family or Order names were available, we assigned those records to ‘species indet.’, ‘Genus indet.’ and ‘Family indet.’. Where there was taxonomic uncertainty, we compiled all plausible taxa names within the same taxonomic level; e.g., the complex *Macropus fuliginosus*/*giganteus*/*titan* comprised *M. fuliginosus* (western grey kangaroo—extant), *M*. *giganteus* (eastern grey kangaroo—extant) and *M*. *titan* (giant kangaroo—extinct). Where Linnaean classifications were discordant among several literature sources or taxa were dubiously identified by researchers, we assigned those records to multiple genera (e.g., *Uromys*/*Melomys*) or species (e.g., *mitchelli*/*minor*). The *FosSahul* database includes a spreadsheet with information regarding taxonomical review (Data Citation 1).

#### Fossil ages

**Figure 2:**
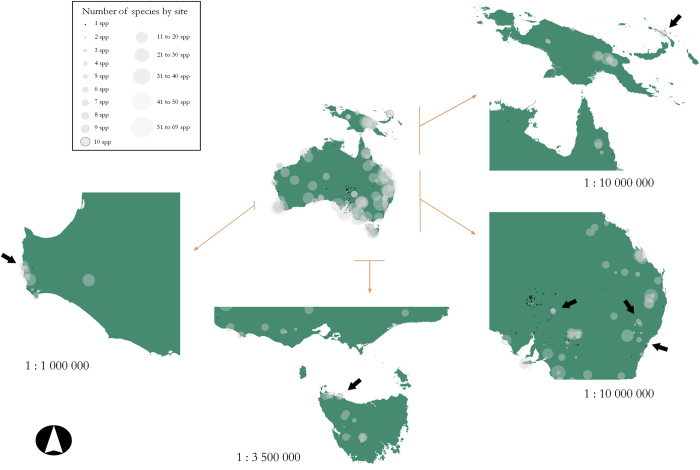
Distribution of cave/site/deposits within Sahul, with proportional circles showing the number of different taxa found per site. Each circle represents a single site. Legend symbol size depends on the scale of each map. Black arrows indicate outlined circles corresponding to sites with 10 species; these circles can be used as a reference scale.

The age of each fossil record includes the label of the dating laboratory, the age estimate with associated uncertainty (e.g., standard deviation), the dated material and the dating technique used (Table 1 (available online only)). Fossils are normally identified and published as part of an assemblage within a cave/site/deposit (Fig. 2), where one or multiple remains/materials were dated to assign an age to a target species. Fossil ages originated from two types of remains: (*i*) fossils—that is, parts of a vertebrate body such as bones, teeth, hair, skin, otoliths or its internally derived products (e.g., gut contents, coprolites, eggshells); and (*ii*) assorted remains, such as artefacts, charcoal, wood, corals, halite crusts, footprints, shells, seeds, sediments and speleothems, which are used to infer the age of the target species based on association (see Table 2). In the same way, dated fossils can provide age estimates for other species’ fossils based on association. Hence, ‘direct’ ages are those derived from the dating of an original component of the fossil of the target species, whereas ‘indirect’ ages are based on dating of associated remains or material.

We assigned single species from a given cave/site/deposit either to a single or to multiple ages (rows in the database) when present in one or multiple depositional contexts (i.e., depth, quadrat, stratum, stratigraphic unit, layer; see Table 1 (available online only)) with associated dated remains.

#### Age reliability

We have developed elsewhere a set of objective criteria to rank the reliability of fossil ages in four categories (A*, A, B, C—from high to low reliability) and, if reliable by association, three sub-categories (w, a, b for ‘within’, ‘above’, ‘below’, respectively)^11^. This quality rating is based on two steps, which we applied to each fossil record in the database. The first criterion (Step 1) is based on the quality of dating protocols, resulting in one of four categories (m*, m, B, C—from high to low reliability). Ages rated as ‘reliable’ (m* and m), if they are indirect ages (see Table 2), pass to the second criterion (Step 2), but if they are direct ages they receive A* or A, respectively, because they do not require an assessment of association (Step 2). Each dating technique and dated material has its own protocols of reliability (Table 3 (available online only)). The second criterion (Step 2) is based on the association between dated materials and fossils of the target species. Only reliable, indirect ages (m* and m) in Step 1 are assessed for association, with three possible outcomes (certain=A, uncertain=B, and equivocal/unknown=C); thus, indirect ages estimated through appropriate, robust dating techniques that have unequivocal association with the fossil remains of the target species can be assigned an A at best. Only direct ages can qualify for the highest quality rating of A*.

For reliable indirect ages, in most cases the fossil remains of the target species and the dated materials are from the same depositional context, and so are assigned to sub-category ‘w’ (within layer). When those depositional contexts differ, ages might still be informative, but should be treated with caution when considered for modelling (e.g., of extinction chronologies). Here, when: (*i*) the fossils are buried above or after (sub-category ‘a’) or below or before (‘b’) the dated material, then those ages do not reflect the target remains’ true age; and (*ii*) the ages are minimum or maximum estimates (*AgeType* sub-field), then the true age of the fossils of the target species can be older or younger than the age of the dated materials, respectively.

#### Geographic location

We gathered information about the geolocation of each deposit when available in the source publication, and we checked for consistency between publications regarding the site where species were recorded (Fig. 2). Decimal approximations in a fossil site’s coordinates were a limitation on the precision of geographic locations (e.g., Noala Rockshelter is indicated as being in the ocean if only two decimal places are provided). When no geolocation was provided in the source publication, we georeferenced locations using GEOLocate software^15^ based on available information. To reduce the chance of encouraging undesirable behaviour at palaeontological/archaeological sites, we also generated our own location uncertainty using the point-radius method to create a circular area around the location. The value in the uncertainty column (Table 1 (available online only)) corresponds to the radius length. Location names were normally given in the source publications, so we maintained the published terminology for the sections or places within a given cave/site/deposit (e.g., stratum, quadrat, stratigraphic unit).

#### General comments

To clarify, refine or complement the metadata associated with individual species records, we collated Supplementary Information that contained additional literature sources, technical aspects or statements published in the source publication or made by the authors of fossil ages related to any field or sub-field of the database (Table 1 (available online only)).

#### Depositional context

We included information regarding the availability of complementary information in the source publication or in any other publication when possible, giving the reference. Additional complementary information relates to the depositional context of the fossil record (i.e., stratigraphy, taphonomy and species abundance), which is valuable for a wider range of uses and analyses (e.g., Bayesian chronological models, understanding past biodiversity commonness and rarity, improvement of species distribution models in palaeo-biogeography).

#### Literature sources

Each fossil record is linked to one literature source, the citation of which includes author(s), year of publication and typical archiving information (e.g., volume, issue, pages, editorial company, publication, place of publication). When a fossil age was published in a source other than that characterizing the entire assemblage of species, we chose the former publication to prevail for citation purposes. We treated all types of literature sources equally, and so we collated unique ages irrespective of source type from research papers and books, government reports and theses. This approach maximized the size of the database, while our quality rating at least guaranteed a robust index of the reliability of age estimates.

## Data Records

The *FosSahul* database is stored as an Excel workbook (Data Citation 1) and structured so that each row contains the age and associated metadata for a single and unique record, with a specific provenance within a given cave/site/deposit. The workbook consists of three worksheets: (1) Main Database, (2) Taxonomic Information, and (3) Literature Sources. In the Main Database, single ages are often used to date multiple species’ records when the dated materials are related to several fossils of identical provenance. Further, ‘na’ indicates missing or unavailable data, and ‘null’ indicates that the field is inapplicable to the content of the corresponding column or sub-field.

*FosSahul* contains 9,302 dated records of fossil vertebrate species from Sahul, including both extant and extinct species (1,957 from extinct species). A total of 478 different species were classified into 215 different genera, while 875 (9%) of the records could be allocated only to the upper taxonomic levels of Family to Order. The database covers 363 caves/sites/deposits corresponding with 351 geographic positions, of which 22% included only one described taxon and 54% included ≤5 taxa (Fig. 2). The database is composed of 144 literature sources with (mainly) a biogeographical, ecological, palaeontological and/or archaeological scope.

## Technical Validation

*FosSahul*’s information is derived mainly from published articles that have already been peer-reviewed. We also did a comprehensive check to remove duplicate records and other errors. We confirmed dubious information and questioned article authors and/or other experts as part of the record-validation process. In addition, our database includes a quality rating of the ages of the fossils, as noted above, which represents the main quality-related validation process for the use of the information.

**Figure 3:**
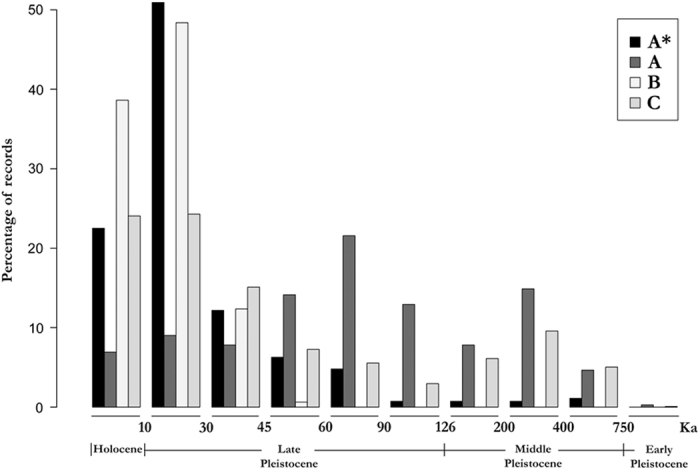
Percentage of records within each category of quality rating for various intervals of time. Holocene (approximately the last 10 thousand years [ka] before present), Late Pleistocene (10 to 126 ka), Middle Pleistocene (126 to 750 ka) and Early Pleistocene (older than 750 ka). Four different ratings are shown: A*/A=high-quality ages and B/C=low-quality ages.

Regarding the quality of such ages (Fig. 3), 271 records (2.9%) had an ‘A*’ rating, 2,151 records (23.1%) were ‘A’ rating, 2,985 records (32.0%) were ‘B’, and 3,895 records (41.8%) were ‘C’. Thus, only 26% of the records are demonstrably reliable (i.e., A* and A categories). Although 54% of the dated species records fall within the last 30 thousand years (ka), 65% of the unreliable ages (B+C categories) are younger than this age. In contrast, 54% of the fossil ages older than 60 ka are reliable (mainly category A). Even with fewer dated fossils in the Early Pleistocene, these records are more reliably dated than many of the more recent fossils (Fig. 3).

## Usage Notes

All fossil records included in the database constitute valuable information on each taxon’s spatial palaeo-distribution, which is obviously unaffected by the age-reliability assessment. We emphasize that *FosSahul* is a ‘living database’ that is open to improvement and updates, resulting from new age estimates being published, and from ages already in our database that have been revisited in the light of improved dating protocols and novel contextual information (e.g., the certainty of association between the fossil remains of target species and the dated materials^11^) (Fig. 1). To make *FosSahul* a centralized archive and repository that facilitates integration, synthesis and an improved understanding of the Sahul fossil record, and to promote information sharing and collaboration, we encourage potential users to provide feedback on the database itself or about new inputs on published and/or unpublished information updates.

## Additional Information

**How to cite this article:** Rodríguez-Rey, M. *et al.* A comprehensive database of quality-rated fossil ages for Sahul’s Quaternary vertebrates. *Sci. Data* 3:160053 doi: 10.1038/sdata.2016.53 (2016).

## Supplementary Material



## Figures and Tables

**Table 1:** Description of fields and sub-fields of information linked to individual records of fossil ages in the *FosSahul* database

**Field**	**Sub-field**	**Description**
ID		Identification number unique for each record
*Geographic position*	*Latitude*	Latitudinal coordinate in decimal degrees—WGS-84 datum
	*Longitude*	Longitudinal coordinate in decimal degrees—WGS-84 datum
	*Uncertainty*	Locality uncertainty obtained from a point-radius method. Units are in metres.
*Linnaean classification*	*Genus*	Most updated genus name
	*Species*	Most updated species name
	*Family*	Most updated family name
	*Class*	Most updated class name
	*Infra-Class*	Most updated infra-class name
	*Order*	Most updated order name
	*Status*	‘Extant’ or ’Extinct’
	*Megafauna*	‘Yes’ if species weight > 44 kg, otherwise ‘No’
*Fossil age*	*AgeID*	Laboratory label or ID given by source publication
	*AgeComments*	Additional information useful to characterize a fossil’s age
	*DatedRemain*	Type of dated remains (e.g., bone, eggshell, sediment, flowstone)
	*DatedMaterial*	Type of dated material (e.g., collagen, carbonate, charcoal, calcite)
	*Age*	Fossil age estimate—sometimes termed ‘date’. In thousands of years (ka)
	*AgeType*	Minimum (‘>’), maximum (‘<’) or exact age (‘=’)
	*Precision*	Dating estimated error of fossil age. In thousands of years (ka)
	*PrecisionType*	Whether the error is reported as ± 1 or 2 standard deviations
	*DatingTechnique*	Method applied to estimate the age of the dated remains/material. *radiocarbon*=beta-counting radiocarbon; *AMS radiocarbon*=accelerator mass spectrometry radiocarbon; *AAR*=amino acid racemization; *U-series Th/U*=uranium series; *ESR*=electron spin resonance; *EU-ESR*=early U-uptake ESR; *LU-ESR*=linear U-uptake ESR; *US-ESR*=coupled U-series and ESR model; *CSUS-ESR*=closed-system U-series and ESR model; *TL*=thermoluminescence; *OSL*=optical stimulated luminescence. *(ABA)* and *(ABOX)* along with *radiocarbon* dating=acid-base-acid and acid-base oxidation pre-treatments, respectively. *(C13 adjusted)* along with *radiocarbon* dating has a -25 o/oo VPBD (Vienna Pee Dee Belemnite) correction and *(SC)*=stepped combustion. *(ICP-MS)* and *(TIMS)* along with U-series Th/U=laser-ablation inductively coupled plasma-mass spectrometry and thermal ionization mass spectrometry, respectively.
	*Calibration*	Whether the published radiocarbon age is ‘calibrated’ or ‘uncalibrated’
	*Association*	Whether the age is ‘direct’or ‘indirect’. See Table 2 for definitions.
*Age reliability*	*Quality category*	‘A*’ (highly reliable), A (reliable), B (unreliable), C (highly unreliable). See main text and Rodríguez-Rey *et al.* ^11^
	*Quality sub-category*	‘a’ (above layer or depositional context or deposited after the dated remain), ‘b’ (below depositional context or deposited before the dated remains), ‘w’ (within layer or same depositional context as that of dated remain). Sub-categories for reliable indirect ages (see main text and Rodríguez-Rey *et al.* ^11^)
*Location*	*OverallRegion*	‘Continental Australia’, ‘Australian Islands’ or ‘New Guinea’
	*AdministrativeDivision*	State, territory or province within overall region
	*SpecificRegion*	Region within administrative division
	*Cave/Site/Deposit*	Name of study location
	*Chamber/Provenance*	Depth, quadrat, stratum, stratigraphic unit and/or layer containing dated fossils
*General comments*		Technical information, additional references or authors’ statements related to the database’s fields and sub-fields
*Depositional context*	*Stratigraphy/Taphonomy*	‘Yes’ if information available in source publication, otherwise ‘No’. ‘Stratigraphy’or ‘Taphonomy’ where only stratigraphic or taphonomic information is available.
	*Species abundances*	Relative abundances available (‘Yes’) or unavailable (‘No’) in source publication
*Literature source*	*Year*	Year of publication
	*Authors*	Authors’ initials
	*Title*	Title of publication
	*Outlet*	Name of publication
	*Archives*	Volume, issue, pages of the publication, and/or publisher
Cells with ‘null’=no correspondence, ‘na’=information not available, and ‘?’=unknown information. Information within brackets is additional information for the sub-field.		

**Table 2:** Definitions for the database.

***Term***	***Definition***
**Age**	Estimated value of absolute age along with the error bounds that result from dating (e.g., 33± 3 ka). Age is sometime termed ‘date’
**Target species**	Vertebrate taxon to which the age under assessment applies (the taxon in the row)
**Direct ages**	Ages on body remains of the target species. Body remains are part of a vertebrate body (e.g., bones, teeth, hair, skin) or its internally derived products (e.g., gut contents, coprolites, eggshell).
**Indirect ages**	Ages not on remains of the target species but can potentially be used to date the target species based on association.
**Association**	Relationship (e.g., stratigraphic) between the fossil of a target species and the dated remains based on the premise that, if there is no evidence of disturbance, remains buried at the same time have the same age. Sometimes body remains are not available, but an association is given based on other evidence that can be linked to the target species (e.g., teeth marks, footprints).
**Depositional context**	Physical setting of the fossils

**Table 3:** Application of dating criteria (Step 1) from^11^

**Dating technique**	**Dated remain/material**	m*	**m**	**B**	**C**
radiocarbon (^**14**^ **C)** **detection limit=55 ka**	bone collagendentin collagen	- collagen preservation checked with C:N ratio and % N and using ultrafiltration, XAD-2, ninhydrin pre-treatments- dating on individual amino acids and using ultrafiltration, XAD-2, ninhydrin pre-treatments		- ABA, AAA or acid-wash pre-treatments- decalcification but no info about collagen presence on bone or dentine - collagen purification difficulties reported	- mixture of multiple bones or teeth
	wood seeds		- dating of alpha-cellulose isolated from plant remains		
	gut contentscoprolites	- dating of alpha-cellulose isolated from digestive remains			
	corals, shells		- dating of carbonate fraction if outer surfaces removed with mechanical grinding and acid wash, and if X-ray diffraction shows that recrystallization is insignificant	- dating without treatment and x-ray diffraction analysis	
	eggshells	- dating of carbonate fraction with stringent removal of secondary carbonate with grinding and acid etching		- dating without treatment	- organic fraction
	charcoal		- ABOX and chlorate oxidation pre-treatments	- ABA, AAA or acid-wash pre-treatments	
	Inorganic calcite (speleothem, soil carbonate) bulk soil organics	not acceptable	not acceptable	not acceptable	not acceptable
amino acid racemization (AAR) **detection limit=1Ma**	eggshellotolith	- direct date on the target species- absolute age requires demonstrated closed-system behaviourand multiple analyses replicated within low uncertaintiesand calibration using independent dating techniques and models describing racemization kinetics	- direct date on the target species- relative age on demonstrated closed-system materialand multiple analyses are replicated within low uncertainties within a limited geographic region (mean annual temperature range<±1 °C)		- unknown thermal history - burnt materials- no local calibration
	bonetooth	not acceptable	not acceptable	not acceptable	not acceptable
Uranium-series **detection limit=500 ka**	tooth	- combined with ESR dating (see Table 5)	- demonstrated closed-system continuous profiles through tooth with laser-ablation ICP-MS, combined with U-uptake modelling- demonstrated closed-system spot sampling with ICP-MS or TIMS, combined with modelling	- ICP-MS or TIMS without modelling	
	bone		- continuous profiles through bone with laser-ablation ICP-MS, combined with uptake modelling- spot sampling with ICP-MS or TIMS, combined with modelling	- ICP-MS or TIMS without modelling	
	eggshell	- eggshell with stringent removal of secondary carbonate, acid wash, and ICP-MS or TIMS			
	closed-system of no body remains (e.g., speleothems, corals		- ICP-MS or TIMS with a detrital correction		
electron spin resonance (ESR) **detection limit=1 Ma**	tooth enamel	- direct age, combined ESR and closed-system U-series modelling (CSUS-ESR) - ESR ages with low U content in dentine and enamel (model independent); internal dose rate <10% of total dose rate; gamma dose rate measured *in situ*	- direct age, EU and LU ages that are model dependent with a *p*-value derived from a U-series estimate (US-ESR) - ESR ages with low U content in dentine and enamel (model independent); internal dose rate <10% of total dose rate; gamma dose rate assumed from sediment attached to tooth	- early U-uptake model (EU) and linear U-uptake model (LU), with no U-series constraint on the possible history of U-uptake- ESR ages with low U content in dentine and enamel (model independent); internal dose rate >10% of total dose rate	
Luminescence **detection limit=1 Ma**	sediment		- single-grain OSL ages for well-bleached or partially bleached sediments that can be modelled- single-grain OSL, single-aliquot OSL or multi-aliquot TL ages on demonstrated well-bleached sediments or sediments with high likelihood of being fully bleached at deposition. Resetting of the luminescence signal needs to be demonstrated explicitly		- single-grain OSL ages that cannot be modelled- single-aliquot OSL or multi-aliquot TL ages for mixed or partially-bleached sediments
	organic material (e.g., bone)	not acceptable	not acceptable	not acceptable	not acceptable
